# Rapid Detection and Trapping of Extracellular Vesicles by Electrokinetic Concentration for Liquid Biopsy on Chip

**DOI:** 10.3390/mi9060306

**Published:** 2018-06-19

**Authors:** Lucia S. Cheung, Sarah Sahloul, Ajymurat Orozaliev, Yong-Ak Song

**Affiliations:** 1 Division of Engineering, New York University Abu Dhabi, PO Box 129188, Abu Dhabi, UAE; lsc6@nyu.edu (L.S.C.); sms30@nyu.edu (S.S.); ajymurat.orozaliev@nyu.edu (A.O.); 2 Department of Chemical and Biomolecular Engineering, New York University Tandon School of Engineering, Brooklyn, NY 11201, USA

**Keywords:** extracellular vesicles, exosomes, biomarkers, liquid biopsy, electrokinetic concentration, ion concentration polarization

## Abstract

Exosomes have gained immense importance since their proteomic and genetic contents could potentially be used for disease diagnostics, monitoring of cancer progression, metastasis, and drug efficacy. However, establishing the clinical utility of exosomes has been restricted due to small sizes and high sample loss from extensive sample preparation. Sample loss is particularly critical for body fluids limited in volume and difficult to access, e.g., cerebrospinal fluid. We present a microfluidic technique that locally enhances the concentration of extracellular vesicles extracted from MDA-MB-231 human breast cancer cell lines by using an ion concentration polarization (ICP)-based electrokinetic concentrator. Our design incorporates a trapping mechanism near the conductive polymer membrane; therefore, we can preconcentrate and capture extracellular vesicles simultaneously. Compared with standard fluorescence detection, our method increased the limit of detection (LOD) of extracellular vesicles by two orders of magnitude in 30 min. Our concentrator increased the extracellular vesicle concentration for 5.0 × 10^7^ particles/1 mL (LOD), 5.0 × 10^8^ particles/1 mL, and 5.0 × 10^9^ particles/1 mL by ~100-fold each within 30 min using 45 V. This study demonstrates an alternative platform to simultaneously preconcentrate and capture extracellular vesicles that can be incorporated as part of a liquid biopsy-on-a-chip system for the detection of exosomal biomarkers and analysis of their contents for early cancer diagnosis.

## 1. Introduction

Tissue biopsy is often hampered by the fact that tumors are localized in areas of the body that are difficult to reach and even surgically inaccessible, making the detection of molecular biomarkers on tumor cells impractical for routine clinical monitoring or for disease diagnostics [1]. In recent years, exosomes have gained immense research interest owing to the significant role they play in orchestrating intercellular communication and molecular exchange [2]. Exosomes are a class of membranous extracellular vesicles (EVs) that originate from inward budding of the endosomal compartment with a cell, forming a multivesicular body that subsequently fuses with the plasma membrane to release the contents [3]. Since they are secreted from virtually all biological fluids (including blood, saliva, urine, synovial, and cerebrospinal fluids [4]), they provide biomarkers indicative of cancer for diagnostic and prognostic purposes [5]. These biomarkers include lipids, proteins, functional messenger RNAs, microRNAs, and double-stranded DNA from their cells of origin [6]. 

The use of exosomes for biomarker analysis requires first and foremost precise separation and purification of exosomes from complex biological fluids. A key challenge continues to be a lack of standardized and efficient methods for separating and purifying exosomes, especially for the separation and purification of body fluid exosomes [7,8,9]. Ultracentrifugation is currently the gold standard for exosome separation; it involves a sequence of centrifugation steps at progressively higher spin speeds of 100,000 rpm or greater to purify exosomes from protein contaminates [10]. This method is time-consuming, requiring four to six hours of processing time by a skilled lab technician. The separated exosomes are frequently contaminated with other proteins and particulates from the medium and cell debris, thus resulting in low recovery and low specificity [11,12]. 

An alternative to ultracentrifugation is a commercial precipitation technology such as Exo-spin^TM^, ExoQuick^TM^, exoEasy Maxi kit, or PureExo^®^ Exosome Isolation kit. These commercial products use special reagents such as polymeric additives to isolate exosomes within ~30 min using a standard centrifuge. While these commercial products are easy to use without expensive ultracentrifuge or advanced technical know-how, the major drawbacks are the proprietary reagents, which may lead to discrepancies in their results [9]. In addition, the reagents can inhibit the recovery of intact exosomes, which could influence the biological activities and characteristics of exosomes [13]. 

Another conventional exosome isolation technology is the immunoaffinity-based approach, which utilizes antibody-coated magnetic beads to capture exosomes that contain specific markers in bodily fluids. This method allows for a specific subpopulation of exosomes to be isolated, but is generally not suited to isolating exosomes from large quantities of biological samples. The removal process of the magnetic beads from exosomes can also be cumbersome [10].

A variety of microfluidic systems for exosome isolation, detection, and analysis has also been reported. These microfluidic platforms differ in terms of yield, sample volume, throughput, fabrication, and operational complexity. Some of the pioneering work on immunoaffinity capture includes Chen et al., who used herringbone groves to increase the capture efficiency in a straight flow surface-modified channel while achieving relatively high throughput and good recovery yield [14]. Dudani et al. developed a microfluidic platform based on rapid inertial solution exchange that facilitated continuous-flow, high-throughput, and 100% transfer efficiency of exosome capture beads from biofluids into a wash buffer [15]. Zhao et al. implemented passive continuous-flow mixing of serum with immunomagnetic beads in a serpentine channel, achieving good recovery of exosomes and enabling bead retention by a magnet in a downstream detection chamber [16]. Reátegui et al. developed the ^EV^HB-Chip, a high-throughput platform that integrates a 3D herringbone microfluidic chaotic mixer with a nanostructured substrate for immunoaffinity-based capture. The platform is able to release captured tumor EVs from the device surface while preserving their cargo contents and a recovery rate up to 94% of the tumor-specific EVs [17]. Membrane-based filtration that isolates exosomes directly from complex biofluids, i.e., blood, is highly advantageous to eliminate any sample pretreatment, especially for point-of-care (POC) applications. Davies et al. demonstrated two membrane-based filtration approaches capable of isolating exosomes from mouse whole blood; however, recovery of exosomes was low [18]. An electrophoretic technique developed by Cho et al. used a dialysis membrane to capture exosomes from diluted mouse plasma, achieving high throughput and a relatively high yield of 65% in approximately 30 min [19]. A new exosome isolation technique was recently reported by Chang et al. that utilizes microfluidic gel electrophoresis and an ion-selective membrane to simultaneously separate and concentrate exosomes from a continuous flowing sample stream. This method proved capable of isolating more than 70% of the incoming exosomes at 150 µL/h for at least 20 min [20]. 

While current microfluidic technology attempts to address the clinical utility of exosomes, fundamental technical challenges still exist due to their small sizes and high sample loss from the extensive sample preparation required prior to measurement. Sample loss poses a severe challenge to the detection of exosomes in body fluids that are limited in volume and difficult to access, such as cerebrospinal fluid. To address this issue, we present a microfluidic technique that locally enhances the concentration of extracellular vesicles extracted from MDA-MB-231 human breast cancer cell lines using an ion concentration polarization (ICP)-based electrokinetic concentrator. Preconcentrating lipid vesicles in a microfluidic channel has been reported most recently by Kim et al., who used an ICP technique to preconcentrate lipid vesicles 160-fold in a single microfluidic channel [21,22,23,24]. However, the trapping of preconcentrated lipid vesicles in the microchannel for further downstream analysis of their contents has not been demonstrated. To demonstrate rapid detection and subsequent trapping in a single microchannel, we used an ICP-based microfluidic concentrator as in our earlier works [25,26]. By printing an ion-selective conductive polymer, poly (3,4-ethylenedioxythiophene)-poly(styrenesulfonate) (PEDOT:PSS), on the PDMS microchannel directly, thus decoupling the ion-selective membrane from the substrate, our device design allows us to incorporate a passive trapping mechanism on the substrate near the conductive polymer membrane; therefore, we can preconcentrate and capture extracellular vesicles simultaneously. Furthermore, our decoupling strategy allows substrate surface modification with standard surface chemistry to capture extracellular vesicles based on aldehyde or antigen–antibody binding, which can result in increased capture specificity.

Compared with standard fluorescence detection, our method increased the limit of detection (LOD) of extracellular vesicles by two orders of magnitude in 30 min. Our concentrator increased the extracellular vesicle concentration for 5.0 × 10^7^ particles/1 mL (LOD), 5.0 × 10^8^ particles/1 mL, and 5.0 × 10^9^ particles/1 mL by ~100-fold for each concentration within 30 min at 45 V/cm. This study demonstrates a feasible alternative platform to simultaneously preconcentrate and capture extracellular vesicles, which can be incorporated as part of a liquid biopsy-on-a-chip system for downstream analysis of exosomal molecular biomarkers such as microRNAs for early cancer diagnosis. 

## 2. Materials and Methods

### 2.1. Device Fabrication and Assembly

**Figure 1:**
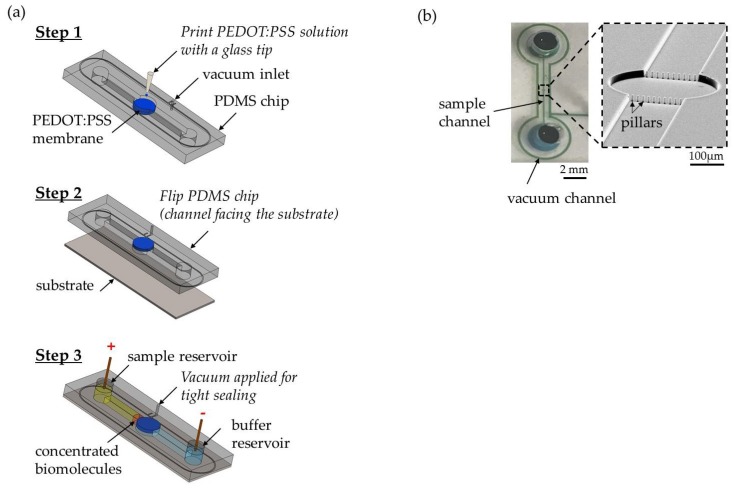
(**a**) Fabrication and assembly of the microfluidic concentrator chip. Step 1: print PEDOT:PSS on the circular pattern of PDMS device directly. Step 2: flip the chip and reversibly attach the device on the SuperEpoxy 3 glass slide. Step 3: apply vacuum suction to enhance adhesion and electrodes were placed at the sample reservoir and the buffer reservoir for electrical voltage to start ICP. (**b**) A photograph of the fully assembled concentrator showing a single microchannel (17 µm high; 1 cm long; 200 µm wide) in PDMS with a reservoir (4 mm of diameter) at each end and a SEM image of a row of pillars (diameter ~10 µm; height ~17 µm) to confine the printed PEDOT:PSS solution on the PDMS.

A standard soft lithography protocol was applied to fabricate the PDMS microfluidic device. As presented in Figure 1a (step 1), we built a single concentrator device by printing a conductive polymer poly(3,4-ethylenedioxythiophene)-poly(styrenesulfonate) (PEDOT:PSS) using GIX microplotter II (Sonoplot Inc., Middleton, WI, USA) on a PDMS device that works as a cation-selective membrane. The PEDOT:PSS circular layer was ~3.0 µm thick and 400 µm in diameter. A row of pillars was used to confine the printed PEDOT:PSS to the PDMS channel. Prior to printing, the device was treated with 1% BSA for an hour to avoid nonspecific binding of the sample. As shown in Figure 1a (step 2), the device was sealed on a SuperEpoxy 3 microarray slide (Arrayit, Sunnyvale, CA, USA) that is commonly used for antibody immobilization. Using this specifically surface-treated microarray slide, we tested the antibody-based detection of extracellular vesicles with surface-printed antibody as capture molecules. To fabricate the antibody microarrays, 10 µg/mL of anti-CD63-biotin (Adipogen AG, Liestal, Switzerland) in 1X PBS buffer solution was printed using a GIX microplotter II and a micro glass tip with a diameter of 40 µm (Sonoplot Inc.). After printing, slides were stored in a petri dish at <50% humidity for 24 h to allow coupling. The glass slides were then treated with two chemicals from protein microarray buffer kit (Arrayit). First, the slide was washed with Arrayit Chemblock™ for 10 min on an orbital shaker at 350 rpm. Then, the slide was rinsed with Arrayit washing buffer for 5 min on an orbital shaker at 350 rpm. The two washing steps deactivated the epoxy surface to enhance sample flowing into the channel and enabled high analytical sensitivity. A second microchannel around the main channel was fabricated, as shown in Figure 1a (step 1), to allow the application of a negative suction to maintain the adherence of the device to the glass slide throughout the experiment. A photograph of the fully assembled concentrator and a SEM image of a row of pillars is shown in Figure 1b. The distance between adjacent micropillars was 10 µm. The purpose of the pillars was mainly to confine the printed PEDOT:PSS solution to the circular pattern on the PDMS, preventing it from flowing into the sample channel. Our test sample in this study was purified extracellular vesicles extracted from breast cancer cell lines rather than from whole blood. Hence, there were no red blood cells in the test sample. It would be feasible to use such an array of micropillars with a smaller gap size to filter out red blood cells in case a whole blood sample is directly used. By printing an ion-selective membrane on the PDMS microchannel directly, we decoupled the ion-selective membrane from the substrate, thus enabling our device design to incorporate any trapping mechanism on the substrate near the conductive polymer membrane to preconcentrate and capture extracellular vesicles simultaneously. To demonstrate this flexibility of interfacing witha variety of substrates, we also used superaldehyde glass substrate (Arrayit) as well as plain glass slide with 3D-printed microtraps to passively trap extracellular vesicles near the ion-selective membrane during ICP at 45 V/cm within 30 min. The traps (a semicircle of 2 μm in diameter was inscribed in a 2 µm × 4 µm rectangle at a height of ~4 µm.) were fabricated on a fused silica substrate using a two-photon polymerization lithography-based 3D printer (Nanoscribe GmbH, Stutensee, Germany). The choice of polymer was IP-DIP by Nanoscribe GmbH. 

### 2.2. Experiment Setup and Data Analysis

For all experiments, an EV sample volume of 30 µL was used, accompanied by 0.05% Tween 20 to avoid EV aggregation. Following the device assembly and before loading any buffer and EV sample into the reservoirs, a syringe pump was used to apply a vacuum suction to enhance adhesion between the PDMS device and the glass substrate. The vacuum suction remained activated during the experiment. The vacuum suction replaced oxygen plasma for PDMS–glass bonding to minimize any effects oxygen plasma may have on the antibodies printed on the glass substrate. As shown in Figure 1a (step 3), 30 µL of 1X PBS buffer solution was loaded into the cathodic reservoir and allowed to flow through the channel using negative suction from the opposite reservoir. The buffer solution was left for 15 min to equilibrate the conductive polymer layer. Next, 30 µL of EV sample was loaded into the anodic reservoir. Finally, an additional 30 µL of buffer was added into the cathodic reservoir to generate a pressure-driven flow of the buffer solution into the sample reservoir that was used for the washing step after preconcentration when the voltage was switched off. The voltage difference in the microchannel generated an electroosmotic flow (EOF) in the direction opposite to the pressure-driven flow and transported the extracellular vesicles towards the conductive polymer layer. Since PEDOT:PSS was negatively charged, it acted as a cation-selective membrane that conducted cations away and thus generated an ion depletion region in front of the conductive polymer layer, which in combination with EOF carrying the extracellular vesicles led to the formation of a plug with enhanced concentration of extracellular vesicles. Even though electrophoretic force was acting on extracellular vesicles with negative surface charge, forcing them to migrate towards the anode, stronger EOF displaced them in the reverse direction towards the cathode. The assembled device was placed on an inverted Nikon Ti-E microscope equipped with a CCD camera (iXON 897, Andor, Belfast, UK). All videos were taken with 20X objective for 30 min using a Cy5 filter to measure the fluorescence signal intensity of the labeled extracellular vesicles with a 1 s exposure time. To start ICP in the device, an electrical voltage of 45 V, 55 V, and 65 V was applied through Pt wire electrodes connected to the DC source meter (Keithley 2400, Tektronix, Beaverton, OR, USA) using the following EV concentrations: 5.0 × 10^7^ particles/1 mL, 5.0 × 10^8^ particles/1 mL, and 5.0 × 10^9^ particles/1 mL. Fluorescence intensity was analyzed using NIH Image J. To quantify the EV concentration, we defined the brightest spot in the plug in front of the PEDOT:PSS membrane as the plug began to form. We followed and measured the spot’s fluorescence intensity with a time interval of one minute during the 30-min concentrating period. The net fluorescence signal intensity emitted from the extracellular vesicle samples was obtained from subtracting the background signal from the channel including that of the remaining free fluorescent dye after four washing steps, as described in Section 2.3. 

### 2.3. Extracellular Vesicles Extraction, Quantification, and Labeling

Breast cancer cell line MDA-MB-231 (Sigma Aldrich, St. Louis, MO, USA) was cultured in DMEM HG Dulbecco’s Modified Eagle’s Medium (D5671, Sigma Aldrich) supplemented with 10% FBS depleted of extracellular vesicles (Thermo Fisher Scientific, Waltham, MA, USA), and Penicillin–Streptomycin (Sigma Aldrich). Cells were cultured until 70–90% confluency and medium collected after 48 h of incubation. Early passages used for all the experiments ranged between passages 7 and 12. Following the exosome extraction protocol provided by an exoEasy Maxi Kit (Qiagen, Hilden, Germany), the medium was filtered after collection with a 0.45 µm cellulose acetate sterile syringe filter (VWR) to remove the cells. Following the extraction, extracellular vesicles were stored in 1X PBS buffer solution at −80 °C until usage. 

To quantify the extracellular vesicles’ concentration after extraction, an EXOCET Exosome Quantification Kit (EXOCET96A-1, System Biosciences, Palo Alto, CA, USA) was used (see Figure S1 showing the standard curve obtained with the kit). The measurement was based on an enzymatic reaction of esterase known to be found in extracellular vesicles from all mammalian species. The EXOCET standards were prepared through serial dilution according to the protocol. To evaluate the signal, a spectrophotometric plate reader (Varioskan Flash, Thermo Fisher Scientific, Waltham, MA, USA) was used at 405 nm. ExoGlow^TM^ protein labeling kit (EXOGP100A-1, System Biosciences, Palo Alto, CA, USA) was used to label protein cargo inside the exosomes. 

To assess the number of washes needed to eliminate excess fluorescent dye from the labeling extracellular vesicles using ExoGlow^TM^ protein labeling kit, we tested the free fluorescent dye background noise through concentrating a negative control sample that included only fluorescent dye using ICP, to compare the background signal intensity of one wash to the background signal of four washes. According to the labeling protocol, the dilution factor of each wash was ~33 after one wash and ~132 after four washes. One washing step resulted in a background signal of 509 RFU, while four washing steps resulted in a lower background signal of 43 RFU. These intensity values were clearly overestimated since the assessment method assumed that none of the dye was used for EV labeling. While the protocol of the labeling kit suggested only one wash, we used a conservative approach and carried out a total of four washes after labeling in all the experiments to minimize the amount of free dye remaining after labeling. 

### 2.4. Characterization of Extracellular Vesicles Concentration with Fluorescence Signal Intensity without ICP

For the calibration curve, three different reference EV concentrations were prepared and their fluorescence signal intensities measured inside the microfluidic channel without ICP. A concentration of 5.0 × $10^{9}$ particles/1 mL, 5.0 × $10^{10}$ particles/1 mL and 5.0 × $10^{11}$ particles/1 mL of extracellular vesicles were used supplemented with 0.05% Tween 20. Without ICP, 5.0 × 10^9^ particles/1 mL was the limit of detection (see Figure S2 that shows the net fluorescence intensity of the microchannel filled with labeled extracellular vesicles of known concentrations of 5.0 × $10^{9}$ particles/1 mL, 5.0 × $10^{10}$ particles/1 mL, and 5.0 × $10^{11}$ particles/1 mL). Using NIH Image J, the fluorescence signal intensity was examined in triplicate for each concentration.

### 2.5. Characterization of Extracellular Vesicles with Transmission Electron Microscopy (TEM) 

**Figure 2:**
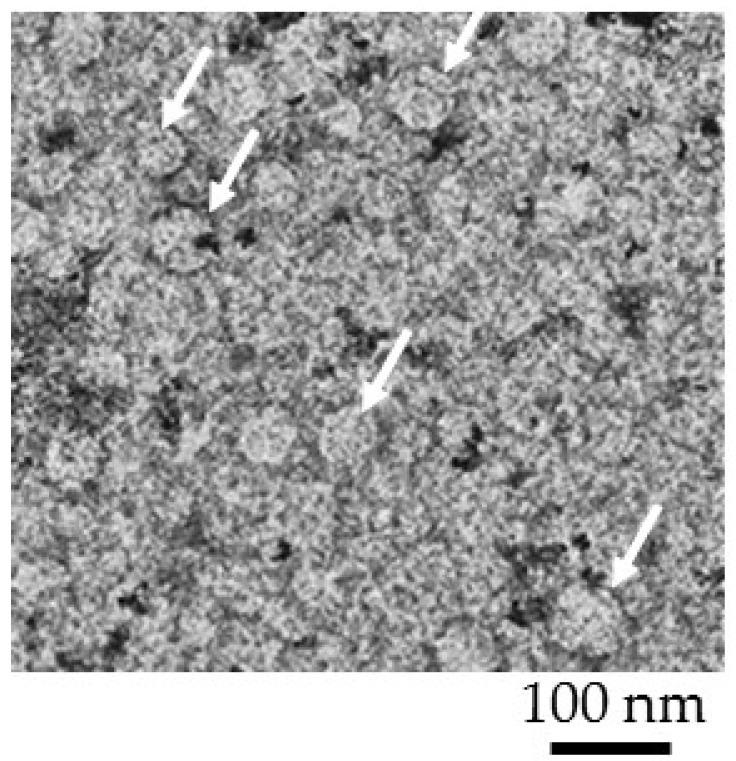
Transmission electron-microscopic observation of extracellular vesicles s at a concentration of 9.0 × 10^7^ particles/1 mL. Average size of an extracellular vesicle was ~50–75 nm. The white arrows pointed to an extracellular vesicle. The Formar carbon copper grid was imaged using FEI Talos F200X Transmission Electron Microscope with a lattice-fringe resolution of 0.14 nm at an accelerating voltage of 200 kV equipped with CETA 16M camera.

Extracellular vesicles with a concentration of 9.0 × $10^{7}$ particles/1 mL were resuspended with 4% paraformaldehyde and fixed on copper girds for 10 min. Following this step, the grids were gently washed with phosphate buffer once and then with distilled water. To stain extracellular vesicles, uranyl acetate substitute dye was used for 5 s. The Formar carbon copper grid was imaged using FEI Talos F200X Transmission Electron Microscope with a lattice-fringe resolution of 0.14 nm at an accelerating voltage of 200 kV equipped with CETA 16M camera. High-resolution images of periodic structures were analyzed using TIA software. EV sizes are shown in Figure 2 with an average size of ~50–75 nm that was in agreement with the reported size of exosomes.

## 3. Results

### 3.1. Evaluating the Enhancement in Concentration of Extracellular Vesicles

**Figure 3:**
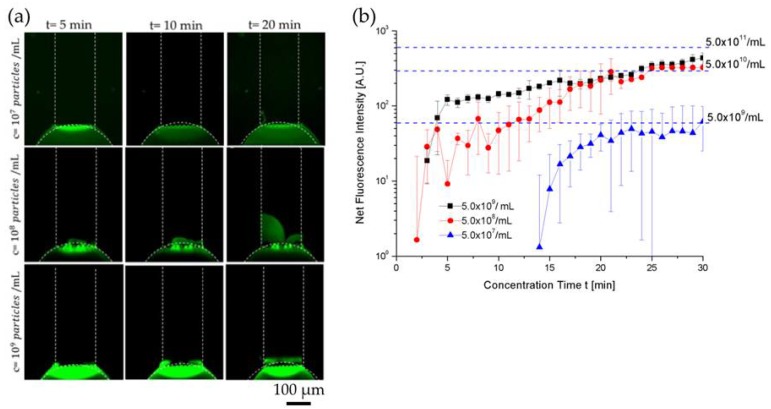
Characterization of extracellular vesicle concentration enhancement for 5.0 × 10^7^ particles/1 mL (LOD), 5.0 × 10^8^ particles/1 mL, and 5.0 × 10^9^ particles/1 mL at 45 V/cm on a SuperEpoxy 3 microarray slide without antibody. (**a**) bright fluorescent plug inside the channel indicating EVs near the conductive polymer membrane—size continued to increase as a function of concentration time. (**b**) Fluorescence signal intensity curves of 5.0 × 10^7^ particles/1 mL (LOD), 5.0 × 10^8^ particles/1 mL, and 5.0 × 10^9^ particles/1 mL. EV concentrations of 5.0 × 10^7^ particles/1 mL (LOD), 5.0 × 10^8^ particles/1 mL, and 5.0 × 10^9^ particles/1 mL increased by ~100-fold within 30 min (ND 1 filter and 1 s exposure time).

At higher electrical fields of 55 V/cm and 65 V/cm, the extracellular vesicle plug was less stable due to strong vortex formation in the depletion zone. At 45 V/cm, a strong fluorescent signal on the ion selective membrane was observed immediately when the voltage was turned on. After 10 min of preconcentration, a bright fluorescent plug was visible inside the channel indicating extracellular vesicles were near the conductive polymer membrane and its size and fluorescence signal intensity continued to increase as a function of concentration time, as shown in Figure 3a. To quantify concentration of extracellular vesicles in the plug, we first measured the fluorescence intensity of the microchannel filled with fluorescently labeled EV samples of known concentrations (5.0 × $10^{9}$ particles/1 mL, 5.0 × $10^{10}$ particles/1 mL, and 5.0 × $10^{11}$ particles/1 mL), as shown in Figure S2, and used them as dotted reference/calibration lines in Figure 3b. We then plotted the fluorescence intensity data as a function of concentration time during ICP electrokinetic preconcentration. To calculate the concentration factor, we estimated the final concentration of extracellular vesicles obtained after 30 min by comparing the fluorescence intensity of EV plug against the reference lines that correspond to the fluorescence intensity values of different EV concentrations. Within 30 min, EV concentration for 5.0 × 10^7^ particles/1 mL, 5.0 × 10^8^ particles/1 mL, and 5.0 × 10^9^ particles/1 mL increased by ~100-fold for each concentration, as shown in Figure 3b. Our limit of detection with ICP was 5.0 × 10^7^ particles/1 mL. Video S1 in the Supplementary Materials shows the EV concentration during ICP at 45 V/cm for 5.0 × 10^9^ particles/1 mL. 

The relatively large error bars in Figure 3b can be explained as follows: (1) EV preconcentration using ICP was a dynamic nonlinear process that was highly dependent on the thickness of the PEDOT:PSS membrane. Because of the inherent variation of the membrane thickness during printing, the preconcentration performance changed from device to device. To minimize this variability, we improved the printing process so that the thickness of PEDOT:PSS circular membrane layer remained at ~3.0 µm. (2) Like all our other preconcentration experiments using ICP, we observed that it normally took approximately 5–10 min of the experiment to stabilize the ICP-based preconcentration plug. For this reason, during the initial 5–10 min of ICP, the error bars were significantly higher than after 10 min, as shown in Figure 3b.

### 3.2. Capturing of Preconcentrated Extracellular Vesicles on an Aldehyde Substrate for Subsequent Imaging and Analysis

**Figure 4:**
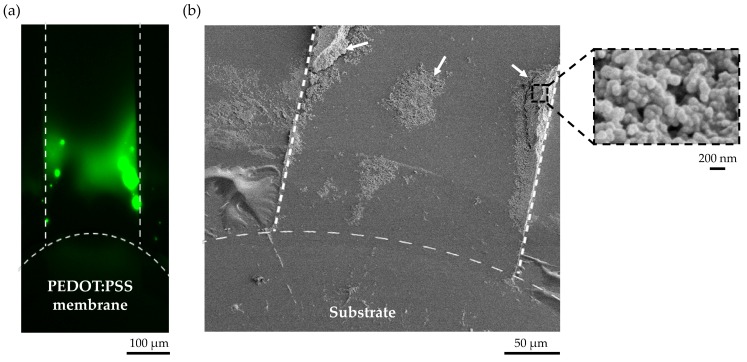
Captured extracellular vesicles on a superaldehyde glass based on surface binding after preconcentration at 45 V/cm for 30 min. (**a**) Electrokinetic preconcentration of extracellular vesicles in the microchannel, (**b**) The two white arrows and the black dotted square in the SEM image indicated areas of captured extracellular vesicles on a superaldehyde substrate after preconcentration. The SEM inset shows aggregated EVs with a size of <~200 nm.

The current device design in combination with a vacuum line allowed us to use any substrate with a specific surface chemistry for capturing extracellular vesicles. To demonstrate this capability, we used a superaldehyde glass substrate to capture extracellular vesicles based on aldehyde surface binding for imaging or any subsequent analysis. Figure 4a shows the preconcentrated EVs in the microchannel. The captured EVs on a superaldehyde glass slide near the conductive polymer membrane after ICP for 30 min at 45 V/m is shown in Figure 4b. For SEM imaging, the reversibly-bonded PDMS cover was simply removed from the substrate by turning off the air suction. 

### 3.3. Capturing of Preconcentrated Extracellular Vesicles with Antibody Binding Assays on Super Epoxy 3 Substrate

**Figure 5:**
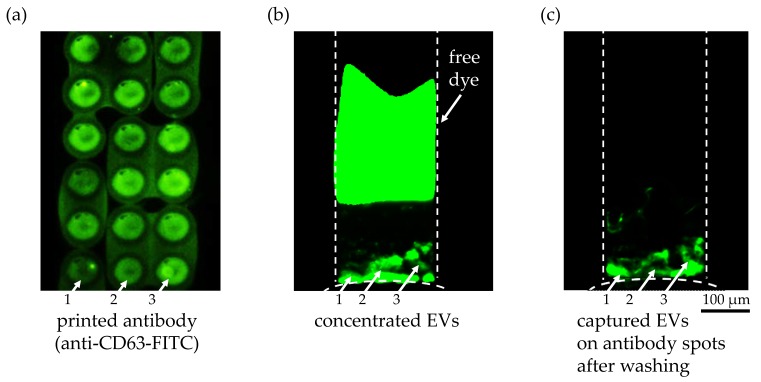
Anti-CD63 antibody on a Super Epoxy 3 slide to capture extracellular vesicles. (**a**) Printed anti-CD63 FITC-labeled antibody after two washing steps to validate the washing protocol. The difference in antibody density within a single spot as well as between spots was clearly visible as indicated with white arrows 1, 2, and 3. (**b**) Fluorescent image of the preconcentrated EVs in the microchannel from an initial concentration of 9.3 × 10^7^ particles/1 mL during ICP for 21 min at 45 V/cm (ND 1 filter and 1 s exposure time). The fluorescently labeled EVs bound on the circular spots of the printed anti CD-63 antibody solution are indicated with white arrows 1, 2 and 3. A second fluorescent plug was visible in the upper region of the microchannel representing the unbound free dye that was separated from the EVs during ICP due to its higher electrophoretic mobility. (**c**) After turning off the voltage at 30 min, the pressure-driven flow from the buffer solution washed away any unbound EVs and free dye into the sample reservoir. The fluorescently labeled EVs bound on the circular spots of the printed anti CD-63 antibody are highlighted with white arrows 1, 2, and 3. The difference in the amount of bound EVs on the antibody spots can be explained by the variation of the antibody density in the spots, as shown in (**a**).

As an alternative capture method with more specificity than the superaldehyde substrate, we printed anti-CD63 antibody on a Super Epoxy 3 microarray slide. First, the surface immobilization and printing quality of the antibody solution were confirmed with the FITC-labeled anti-CD63 antibody, as shown in Figure 5a. Several fluorescent circular spots indicating anti-CD63 antibody were clearly visible after printing on a Super Epoxy substrate and washing, though slightly blurry due to the overlapping of the printed spots. Also, an uneven density of the antibody solution in each spot as well as between spots could be observed, as indicated by white arrows 1, 2, and 3, which might explain the non-uniformity of the EVs bound on antibody spots 1, 2, and 3 after the washing step. The fluorescently labeled EVs bound on the circular spots of the printed anti-CD63 antibody solution were detected during ICP in Figure 5b and after ICP and washing in Figure 5c, as indicated by the white arrows. The fact that the remaining free fluorescent dye after labeling of the EVs after extraction was completely separated from the EV during ICP, as shown in Figure 5b, and did not show any non-specific binding on the printed antibody (Figure 5c) was another encouraging result. It implied that our preconcentration technique could eliminate the time-consuming washing steps to remove unbound free dye after labeling of EVs. The inherent separation process based on the different electrophoretic mobilities of the dye and the EVs during ICP enabled simpler sample preparation of EVs for fluorescence detection. Using this immunoaffinity capture method, we have successfully demonstrated that the captured particles on the substrate were the EVs with CD63 surface marker and that our preconcentration technique enhanced the capture efficiency of EVs while allowing high specificity based on antigen–antibody binding affinity. The negative control experiment without immobilized antibody showed no binding of EVs on the substrate after preconcentration.

### 3.4. Trapping of Preconcentrated Extracellular Vesicles with Microtraps on Glass Substrate

**Figure 6:**
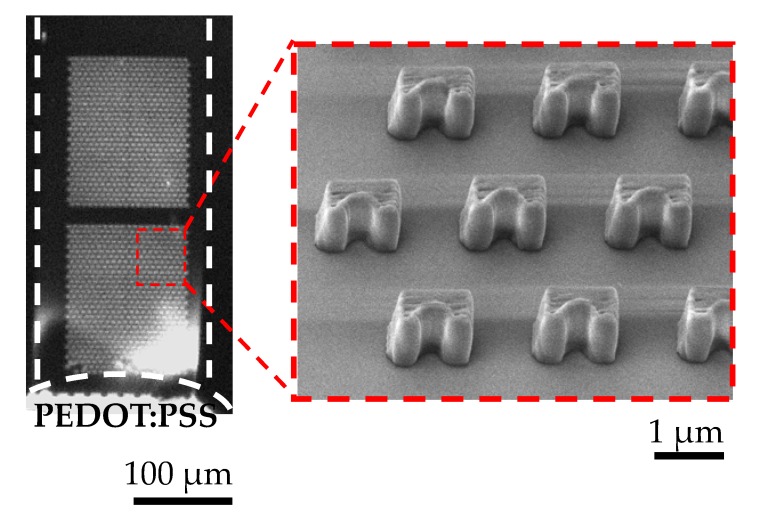
Trapping of preconcentrated extracellular vesicles with the microtraps on a glass surface for an initial concentration of 2.0 × 10^8^ particles/1 mL at 45 V/cm. The microtrap was a semicircle of 2 μm in diameter inscribed in a 4 µm × 2 µm (width × depth) rectangle. The height of the structure was ~4 µm.

Our decoupling strategy of the ion-selective membrane from the substrate even enabled the concentrator device to incorporate a passive trapping mechanism on the substrate near the conductive polymer membrane to capture extracellular vesicles during preconcentration, as shown in Figure 6. Since the main purpose of this study was to demonstrate this capability, the number of rows for the microtraps was chosen arbitrarily. The number of rows will further be optimized to increase the capture efficiency of EVs. It is expected that higher number of rows will result in higher probability of capture. We will further optimize the design of these passive traps by taking into account the deterministic lateral deviation of the EVs’ trajectories. In this way, we expect to significantly increase the capture efficiency.

## 4. Discussion

Compared to fluorescence detection of extracellular vesicles in a microfluidic channel without ICP, our method allowed us to lower the LOD of extracellular vesicles by two orders of magnitude within 30 min. Our concentrator increased extracellular vesicles’ concentration for 5.0 × 10^7^ particles/1 mL (LOD), 5.0 × 10^8^ particles/1 mL, and 5.0 × 10^9^ particles/1 mL by ~100-fold for each concentration within 30 min using an electrical field strength of 45 V/cm. Our limit of detection at 5.0 × 10^7^ particles/1 mL was higher compared to other detection limits reported in the literature such as 7.5 × 10^5^ particles/1 mL in the case of ovarian cancer extracellular vesicles [16] or 2.8 × 10^6^ particles/1 mL in the case of breast cancer extracellular vesicles [27]. It is important to note that the purpose of this study was not to achieve lower LOD compared to the literature values but rather to demonstrate the capability of our preconcentrator device to lower the detection limit with ICP compared to standard detection, whatever the detection method used might be. Further optimization of the ICP-based concentrator device, such as printing a thicker conductive polymer membrane and using a higher conductive grade PEDOT:PSS, could lead to more sensitive detection.

We have demonstrated that, by decoupling the ion-selective membrane from the substrate, it allows the flexibility of either using an antibody-immobilized substrate to increase the capture specificity of extracellular vesicles for downstream analysis or a plain glass substrate with passive microtraps to capture extracellular vesicles in a highly-ordered manner. We envision that, by adding a lysis step after trapping, we can collect the lysate for downstream analysis such as RNA sequencing or any other type of molecular analysis that will be the focus of our future study. 

## 5. Conclusions

We have demonstrated rapid detection and subsequent trapping in a single microchannel using an ICP-based microfluidic preconcentrator. By printing an ion-selective membrane directly on the PDMS microchannel, we have decoupled the ion-selective membrane from the substrate. Using this fabrication strategy in combination with a vacuum suction channel, our device could incorporate a surface binding-based or passive trapping mechanism near the conductive polymer membrane for SEM imaging or any other downstream analysis; therefore, we can simultaneously preconcentrate and capture extracellular vesicles. This new preconcentrator chip might open up new opportunities to explore the full potential of extracellular vesicles as biomarkers for liquid biopsy.

## Supplementary Materials

The following are available online at http://www.mdpi.com/2072-666X/9/6/306/s1. Figure S1 shows the standard curve obtained with the EXOCET Exosome Quantification Kit. Figure S2 is the fluorescence intensity analysis for ND 1 Filter and 1 s exposure time. It shows the net fluorescence intensity of the microchannel filled with labeled extracellular vesicles with three different concentrations of 5.0 × $10^{9}$ particles/1 mL, 5.0 × $10^{10}$ particles/1 mL and 5.0 × $10^{11}$ particles/1 mL. Video S1 shows electrokinetic concentration of EVs during ICP at 45 V/cm starting from an initial concentration of 5.0 × 10^9^ particles/1 mL.
