# Supplementary material for: Rapid Detection and Trapping of Extracellular Vesicles by Electrokinetic Concentration for Liquid Biopsy on Chip
Source: Micromachines (Basel). 2018 Jun 19;9(6):306. doi: 10.3390/mi9060306 (PMC6187315; doi:10.3390/mi9060306)
Supplement: Supplementary file 1 [file micromachines-09-00306-s001.zip › micromachines-304772-SI/micromachines-304772-SI.pdf]

Supplementary Materials: Rapid Detection and Trapping of Extracellular Vesicles by Electrokinetic Concentration for Liquid Biopsy on Chip

Lucia S. Cheung, Sarah Sahloul, Ajymurat Orozaliev and Yong-Ak Song

1. Standard Curve for EXOCET Exosome Quantification Essay

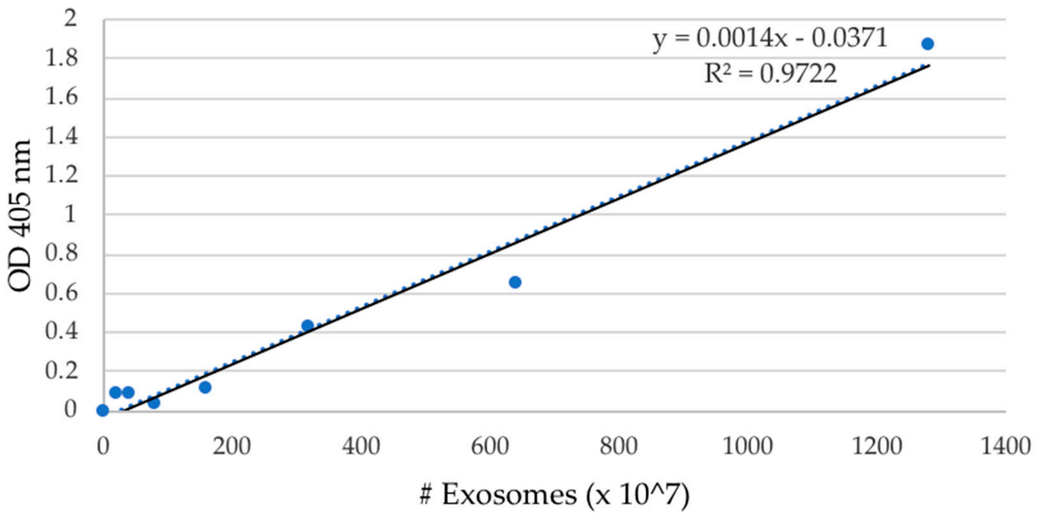

Figure S1. The standard curve provided by the EXOCET assay kit is calibrated to the signal detected from a number of exosomes as measured by NanoSight (NTA) analysis. To quantify our exosomes, the sample readings are plotted on the standard curve.

2. Calibration Curve

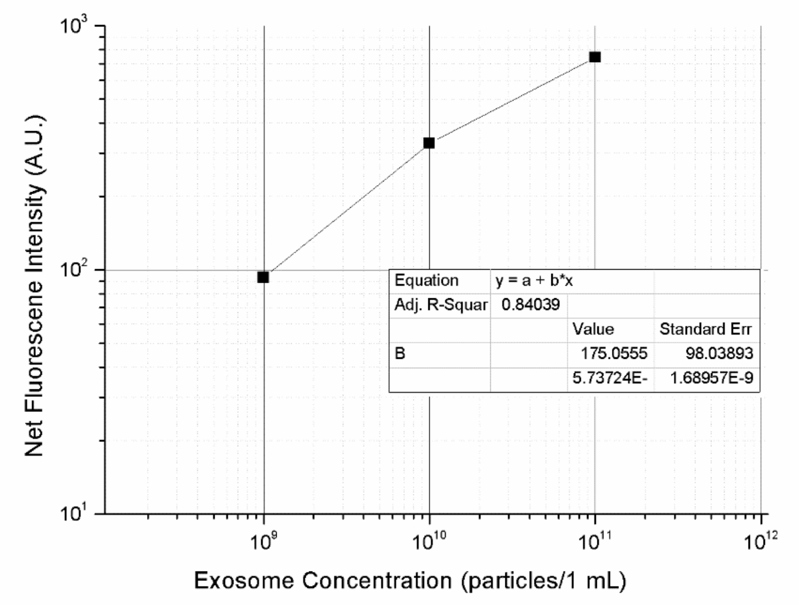

Figure S2. Characterization of exosomes concentration with fluorescence signal intensity without ICP. Net fluorescence intensity of the microchannel filled with labelled exosomes without ICP of known concentrations 5.0×10^9 particles/1 mL, 5.0×10^{10} particles/1 mL and 5.0×10^{11} particles/1 mL. 5.0×10^9particles/1 mL is our detection limit without ICP.

3. Video of Exosome Concentration during ICP

Video S1: This video shows electrokinetic concentration of EVs during ICP at 45 V/cm starting from an initial concentration of 5.0×10^9 particles/1 mL.
